# RHBDF2 is correlated with immune infiltrates in hepatocellular carcinoma and may have potential as a biomarker

**DOI:** 10.1002/2211-5463.13598

**Published:** 2023-04-05

**Authors:** Hanjuan Gong, Hailun Xie, Zhimin Huangfu, Shuxian Zhang, Yi Tang, Ming Xiao, Ming Li, Yalan Wang

**Affiliations:** ^1^ Department of Pathology, Molecular Medicine and Cancer Research Center, Basic Medicine College Chongqing Medical University Yuzhong China

**Keywords:** bioinformatics analysis, hepatocellular carcinoma, immune infiltration, prognostic biomarker, *RHBDF2*

## Abstract

Immune checkpoint inhibitors have been approved for the treatment of advanced hepatocellular carcinoma (HCC). However, immunotherapy requires the identification of suitable biomarkers to guide treatment. The gene for rhomboid 5 homolog 2 (*IRHom2*), which encodes the rhombus protease iRhom2, activates the MAP3K7‐dependent pathway and promotes hepatic steatosis. Thus, we hypothesized an involvement of this gene in HCC. We report that *RHBDF2* expression is dramatically upregulated in HCC. *RHBDF2* upregulation is associated with tumor stage, lymph node metastasis, tumor protein P53 mutation, and worse prognoses in HCC patients. Gene Ontology, Kyoto Encyclopedia of Genes and Genomes and gene set enrichment analysis enrichment analysis indicated that *RHBDF2* was involved in immune signal pathways. Moreover, *RHBDF2* expression was positively related not only to immune infiltration, but also to immune cell markers. Examination of several types of HCC infiltrated by immune cells revealed that the group with high expression of *RHBDF2* showed the worst outcomes. Therefore, *RHBDF2* may have potential as a novel biomarker for predicting prognosis and is related to immune infiltrates in HCC.

## Graphical abstract

The gene for rhomboid 5 homolog 2 (*RHBDF2*) is a protein‐coding gene and participates in important biological processes. We found that overexpression of *RHBDF2* predicts worse prognosis for HCC patients and is significantly associated with immune cell infiltration. Furthermore, *RHBDF2* can impact the prognosis of HCC patients partly because of tumor immune infiltration in HCC.

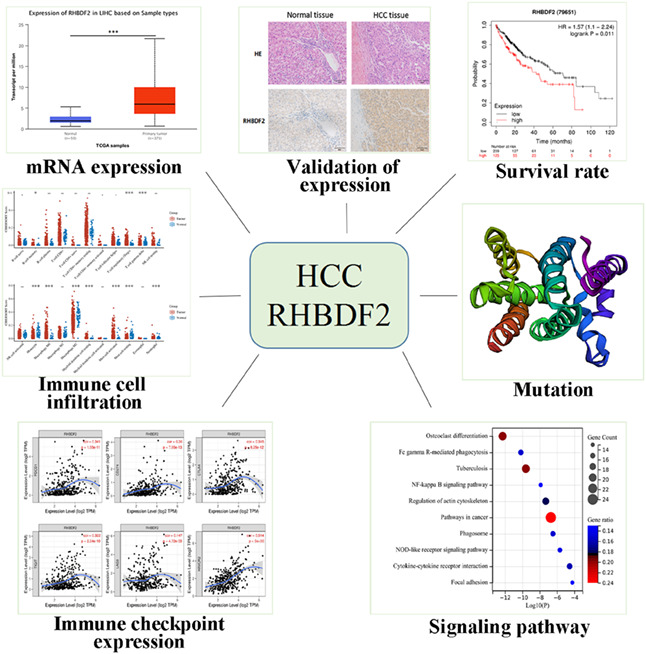

**Abbreviations**
ADAM17: ADAM metallopeptidase domain 17
AJCC: American Joint Committee on Cancer
DFS: disease‐free survival
DSS: disease‐specific survival
EGFR: epidermal growth factor receptor
GO: Gene Ontology
GSEA: gene set enrichment analysis
HCC: hepatocellular carcinoma
HR: hazard ratio
KEGG: Kyoto Encyclopedia of Genes and Genomes
MAP3K7: mitogen‐activated protein kinase 7
MHC: major histocompatibility complex
OS: overall survival
PFS: progression‐free survival
RFS: recurrence‐free survival
RHBDF2: rhomboid 5 homolog 2
TGF: transforming growth factor
TNF: tumor necrosis factor
TP53: tumor protein P53

Hepatocellular carcinoma (HCC), a common primary malignancy, originating from liver cells [1, 2, 3]. It is worth noting that HCC is a common cause of cancer‐related death [4, 5]. Surgical treatment is the first choice in early‐stage HCC patients, and chemotherapeutic drugs, radiotherapies, targeted therapies and immunotherapy are suitable for advanced patients [6, 7, 8, 9]. Regarding the mechanism of inflammation in tumors, immunotherapy as a new therapeutic approach has received increasing attention. Recently, immune checkpoint inhibitors were approved, including Nivolumab, which is an option for advanced HCC patients. However, immune checkpoint inhibitors show a lower objective response rate and survival rate [10, 11, 12]. Thus, there is an urgent need to explore new prognostic biomarker in HCC.

The gene for rhomboid 5 homolog 2 (*RHBDF2*) is a protein‐coding gene that encodes rhombus protease, iRhom2, which participates in important biological processes [13, 14, 15]. *RHBDF2* can be expressed in immune cells, heart and epithelial cells, and has an important impact on the disease by activating the ADAM metallopeptidase domain 17 (ADAM17) signaling pathway [16, 17]. Several recent studies have suggested that *RHBDF2* is significantly upregulated in tumors and has pro‐tumor effects [18, 19, 20]. Molecular mechanism studies have shown that RHBDF2 activates the mitogen‐activated protein kinase 7 (MAP3K7)‐dependent pathway and promotes hepatic steatosis [21]. Furthermore, RHBDF2 activates tumor necrosis factor (TNF)‐α, and epidermal growth factor receptor (EGFR) signaling and enhances lupus nephritis [22]. High *RHBDF2* expression can induce enhanced EGFR, WNT and transforming growth factor (TGF)‐β signaling, and was dramatically linked to poor prognosis of cervical cancer [23]. Additionally, *RHBDF2* overexpression activated TGF‐β signaling and enhanced the aggressiveness of cancer cells in gastric cancer [24]. Recent studies have shown that RHBDF2 has a critical impact on the immune regulation of disease [16, 25]. Therefore, RHBDF2 has a multifaceted influence on the tumor immune microenvironment, which has a pro‐tumor effect. Nevertheless, the potential value and molecular mechanism of RHBDF2 in HCC patients are still unknown.

The present study aimed to evaluate the potential prognoses of RHBDF2 and its biological functions in HCC. Accoridingly, we evaluated *RHBDF2* expression and its association with prognoses using bioinformatics tools. In addition, mutations of *RHBDF2* and the link between RHBDF2 and immune cell infiltration were investigated in HCC. Mechanistically, we mined *RHBDF2* co‐expressed genes and analyzed their biological functions using bioinformatics tools. Importantly, quantitative reverse transcriptase‐PCR, western blotting and immunohistochemistry were applied to detect *RHBDF2* expression in HCC. Our data implied that the high *RHBDF2* expression can predict a worse prognosis and regulate immune cell infiltration in HCC.

## Materials and methods

### Analysis of RHBDF2 expression

We investigated RHBDF2 mRNA levels among 23 types of cancers with the TIMER database (https://cistrome.shinyapps.io/timer). The relative mRNA level of *RHBDF2* in 25 HCC cell lines was analyzed with the CCLE database (https://sites.broadinstitute.org/ccle). Furthermore, the mRNA level of *RHBDF2* was also analyzed with the GEPIA database (http://gepia.cancer‐pku.cn). Additionally, we utilized the UALCAN database (http://ualcan.path.uab.edu/index.html) containing clinical data of various cancers to investigate the mRNA levels of *RHBDF2* in different samples, lymph node metastasis status, tumor protein P53 (TP53) mutation status, tumor grade and tumor stage.

### Cell culture, RNA extraction and quantitative reverse transcriptase‐PCR (RT‐qPCR)

The normal human liver cell line LO2 and human HCC cell lines (Hep G2, SK‐HEP‐1, Hep 3B2.1‐7) were provided by Professor Li Tang (Professor of Chongqing Medical University, Chongqing, China) and maintained in Dulbecco's modified Eagle's medium or RPMI1640 medium (Hyclone, Logan, UT, USA) with fetal bovine serum and penicillin and streptomycin (Beyotime Biotechnology, Shaghai, China). The incubator was set at 37 °C with 5% CO_2_. The Trizol method (Takara, Kusatsu, Japan) was applied for extracting total RNA. The PrimeScript Reverse Transcription Kit (Takara) was employed for the synthesis of cDNA, and then RT‐qPCR was carried out, and beta‐actin expression was used to normalize the results. Beta‐actin (forward primer: GCCCTAGACTTCGAGCAAGA; reverse primer: AGGAAGGAAGGCTGAA GAG), RHBDF2 (forward primer: GATGGGGCAGACACGTTTGA; reverse primer: CCTCGGAAGTAGCTGGCAG) were used for RT‐qPCR.

### Western blotting

After extracting the protein from the cells, we measured the protein concentrations, and then electrophoresed proteins. Subsequently, we transferred proteins to poly(vinylidene difluoride) membranes (Millipore, Burlington, MA, USA). We blocked membranes with non‐fat milk and incubated membranes with RHBDF2 (dilution 1 : 1500; Proteintech Wuhan Sanying, Wuhan, China) and beta‐actin (dilution 1 : 8000; Proteintech Wuhan Sanying) antibody. After washing, a secondary antibody was applied for incubating the membranes. Finally, the blots were detected with BeyoECL plus (Millipore), and the signals were visualized via a Gel Imaging System (Bio‐Rad, Hercules, CA, USA). imagelab (Bio‐Rad) was employed for the analysis of gray values. The experiment was repeated three times.

### Immunohistochemistry staining and hematoxylin and eosin staining

We collected HCC pathological tissue from 40 patients and adjacent normal tissue from 15 cases in the Pathology Department of the First Affiliated Hospital of Chongqing Medical University (Chongqing, China). All patients provided written informed consent, and the study was conducted in accordance with the Declaration of Helsinki. The study passed the ethical review of the Ethics Committee of Chongqing Medical University (permit number: 2022‐K511). Briefly, after dewaxing, all tissue slices were rehydrated, antigen retrieved, and then 3% hydrogen peroxide was applied. Sections were incubated with RHBDF2 (dilution 1 : 200; Proteintech Wuhan Sanying) antibody at 4 °C overnight after the block. A secondary antibody was applied to incubate the sections. Finally, 3,3′‐diaminobenzidine was employed for staining and then sections were counterstained with hematoxylin. Two independent pathologists analyzed immunohistochemistry staining. When a disagreement occurred, a third senior pathologist was attended to make the final diagnosis [26]. After dewaxing, tissue slices were rehydrated and stained with hematoxylin and eosin, and then dehydrated and mounted.

### Prognostic analysis

Kaplan–Meier Plotter (https://kmplot.com/analysis/index.php?p=background), a prognostic analysis database, was applied for investigating the prognosis of HCC patients in high‐ and low‐expression *RHBDF2* groups.

The clinical data for patients with HCC were downloaded from the Sanger box platform (http://vip.sangerbox.com) and then the link between *RHBDF2* and the prognosis was evaluated. In addition to this, the prognostic effect of *RHBDF2* was analyzed for different clinicopathological parameters of HCC. Moreover, the prognosis of patients with *RHBDF2* overexpression based on immune cell infiltration was also explored.

### Mutation analysis

The cBioPortal database (http://www.cbioportal.org), an online cancer genomics dataset, was employed for investigating the mutations of *RHBDF2* in 1045 HCC patients from three datasets through the cBioPortal database. Apart from this, the prognostic values of HCC patients with *RHBDF2* mutations or without *RHBDF2* were no different.

### Gene Ontology (GO), Kyoto Encyclopedia of Genes and Genomes (KEGG) and gene set enrichment analysis (GSEA)

The co‐expressed genes associated with *RHBDF2* were chosen via the LinkedOmics database (http://www.linkedomics.org/login.php) using Pearson' correlation analysis (|*r*| ≥ 0.3). GO (http://geneontology.org) and KEGG (https://www.genome.jp/kegg) analysis was undertaken with the Metascape database (https://metascape.org/). To investigate the pathways based on *RHBDF2* expression, we utilized gsea software (https://www.gsea‐msigdb.org/gsea) to investigate the molecular functions of high RHBDF2 expression in HCC. Normalized ES > 1 is an enrichment standard in GSEA, and statistical significance was considered when *P* < 0.05.

### Immune infiltration

The CIBERSORT database (https://cibersort.stanford.edu) contains immune data about various immune cell infiltration in various human cancers and can provide an estimate of the abundance of 22 tumor‐infiltrating immune cells. We used this tool to examine the fraction of 22 immune cell types in HCC and normal tissues.

To investigate immune infiltration of HCC, we utilized the two latest algorithms, TIMER and Quan Tiseq [27], to measure the immune infiltration levels in 371 HCC patients. Furthermore, we evaluated the link between *RHBDF2* upregulation and immune checkpoint molecules and markers of different immune cell subgroups with the TIMER database.

### Statistical analysis

The GEPIA, UALCAN and TIMER databases were utilized for evaluating *RHBDF2* expression, and the comparisons of RHBDF2 expression between HCC and normal tissues were performed with Student's *t*‐test. Logistic regression was employed for evaluating the prognosis, and Pearson correlation analysis was also employed for evaluating the relationship between RHBDF2 expression and other gene expressions. *P‐*value < 0.05 was considered statistically significant.

## Results

### RHBDF2 expression is upregulated in HCC patients

**Fig. 1:**
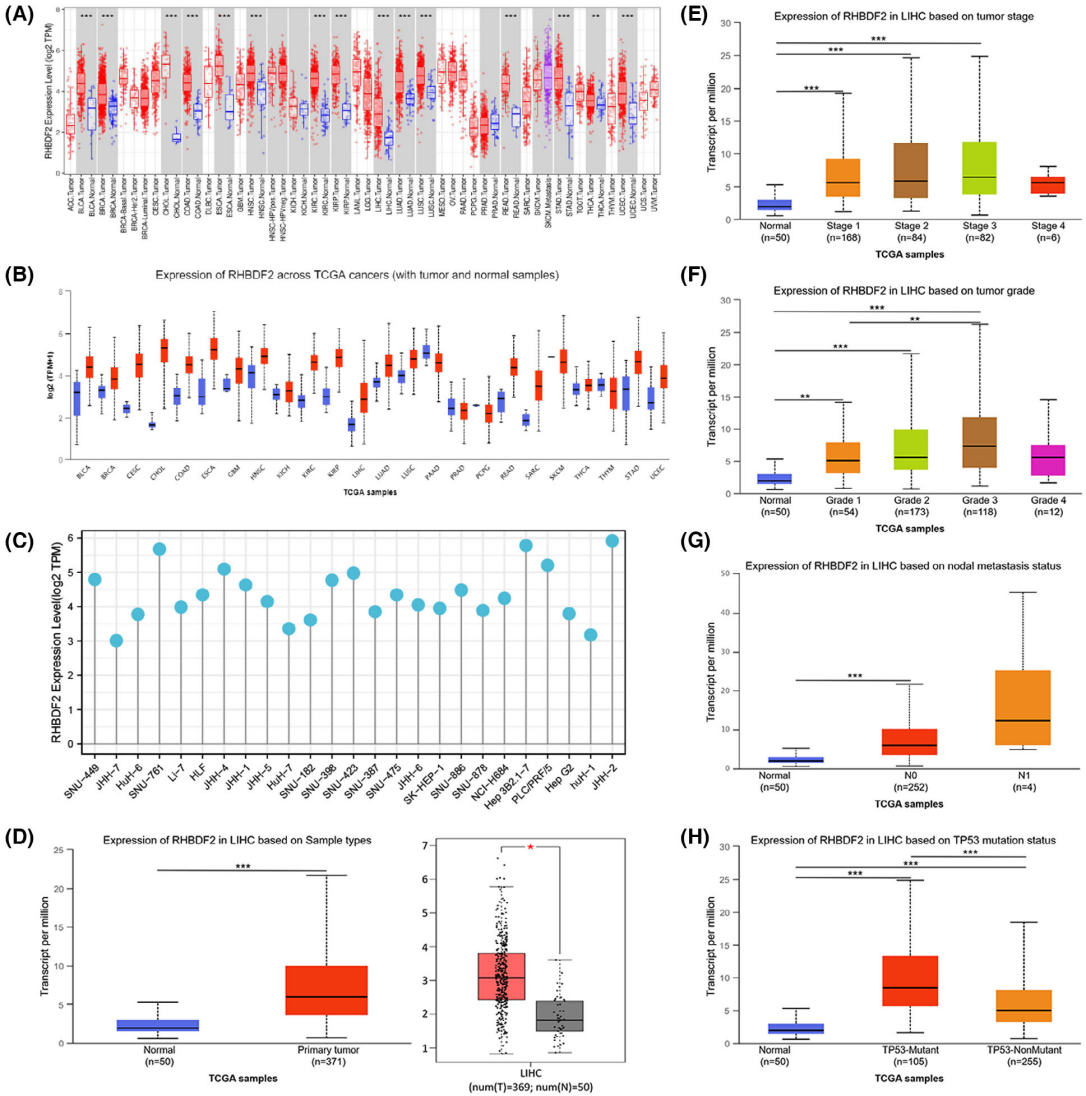
Expression of *RHBDF2* in HCC and its correlation with clinical features of HCC. (A, B) *RHBDF2* expression in pan‐cancer was analyzed using the TIMER database (A) and the UALCAN database (B). (C) mRNA expression of *RHBDF2* in various HCC cell lines was analyzed with the CCLE database. (D) *RHBDF2* expression in HCC and adjacent normal tissues was determined through the GEPIA and UALCAN databases. (E–H) *RHBDF2* mRNA expression was related to HCC patients' stages (E), grade (F), nodal metastasis (G) and TP53 mutation (H). The statistical significance was computed by the Wilcoxon test in the TIMER database and estimated by Student's *t*‐test in the GEPIA and UALCAN databases. *P < 0.05, **P < 0.01, ***P < 0.001.

First, the mRNA level of *RHBDF2* was investigated in human cancers with the TIMER database, and we found that *RHBDF2* mRNA expression was upregulated in most human tumors, including HCC (LIHC; Fig. 1A). The UALCAN database was utilized for further investigation of *RHBDF2* expression in each tumor and corresponding normal tissues (Fig. 1B), which was consistent with the analysis of the TIMER database. Here, *RHBDF2* expression in HCC was the main focus of the research. We observed that *RHBDF2* mRNA expression was higher in HCC tissues than in normal tissues with the GEPIA and UALCAN databases (Fig. 1D). Furthermore, the CCLE database was employed for analyzing *RHBDF2* expression levels of various HCC cell lines (Fig. 1C).

### Association between RHBDF2 expression and clinical parameters in HCC

The UALCAN database was applied for investigating the link between RHBDF2 and the clinicopathological parameters in HCC. According to the tumor stage, *RHBDF2* overexpression in HCC patients in stages 1, 2, and 3 was observed compared to normal liver tissue (Fig. 1E). Based on tumor grade, significant overexpression of *RHBDF2* was observed in HCC patients in grades 1, 2, and 3 compared to normal liver tissue. Importantly, a clear difference in *RHBDF2* upregulation was found in HCC patients in grades 1 and 3 (Fig. 1F). For lymph node metastasis, significant overexpression of *RHBDF2* was found in HCC patients with NO state compared to normal controls (Fig. 1G). Additionally, we found that RHBDF2 expression was upregulated in both TP53 mutant and TP53 wild‐type HCC patients compared to normal controls, whereas *RHBDF2* expression was significantly higher in TP53 mutant HCC patients than in TP53 wild‐type HCC patients (Fig. 1H). The results above indicate that high *RHBDF2* expression remarkably correlated with progression and lymph node metastasis in HCC patients.

### Validation of differential expression of RHBDF2 in HCC

**Fig. 2:**
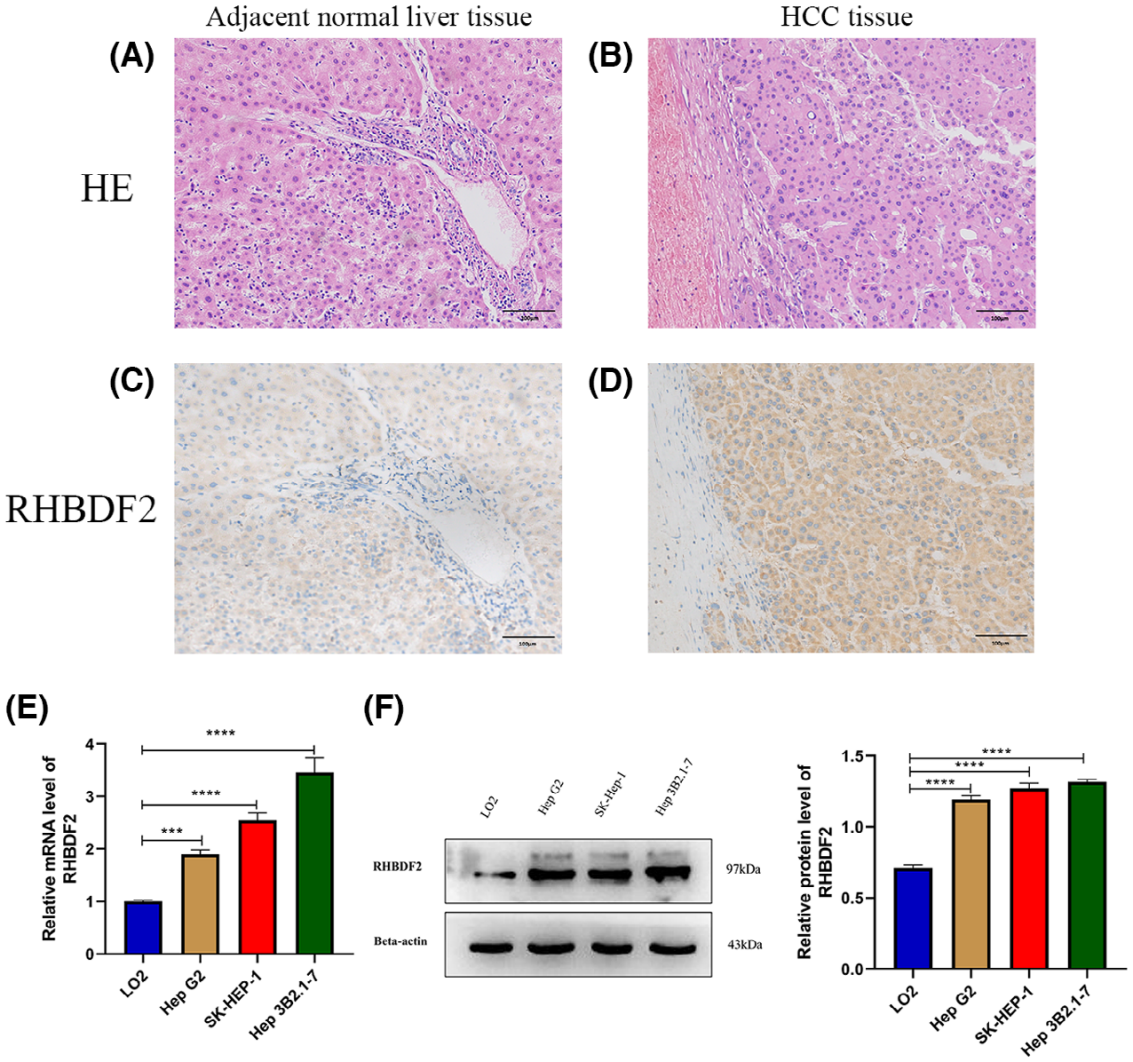
Validation of *RHBDF2* expression in HCC. (A–D) Immunohistochemical staining and hematoxylin and eosin staining of RHBDF2 were performed in HCC tissues (*n* = 40) and normal tissues (*n* = 15). Representative images are shown. Scale bars = 100 μm. RT‐qPCR (E) and Western blotting (F) were used to analyzed the expression of RHBDF2 in four different cell lines. The mean ± SD is shown. Statistical significance was determined using one‐way ANOVA with a post‐hoc Tukey's test. ***P < 0.001, ****P < 0.0001.

To analyze in‐depth protein expression of *RHBDF2* in HCC, protein expression of *RHBDF2* in HCC and normal tissues was examined by immunochemistry staining. *RHBDF2* overexpression was discovered in HCC according to the results of immunochemistry and was mainly expressed in the cytoplasmic compared to normal tissues (Fig. 2C,D). It is worth noting that RHBDF2 expression was linked to the differentiation degree of HCC tissues (*r* = 0.438, *P* = 0.005) (Table 1). The results of hematoxylin and eosin staining in HCC and adjacent tissues are shown in Fig. 2A,B. Additionally, the expression of *RHBDF2* also was examined in several HCC cell lines. We discovered that *RHBDF2* expression is upregulated in HCC cell lines (HepG2, SK‐Hep1, Hep3B2.1‐7) according to the outcomes of RT‐qPCR and western blotting compared to normal liver cell line LO2 (Fig. 2E,F).

**Table 1:** The correlation between the positive degree of *RHBDF2* and differentiation of HCC in tissues (Spearman method).

	Positive degree			Total	Correlation (spearman)	*P*‐value
	+	++	+++			
Differentiation degree						
Well differentiated	0	8	2	10	0.438	0.005
Moderately differentiated	2	11	4	17		
Poorly differentiated	0	3	10	13		
Total	2	23	16	40		

### Correlation between RHBDF2 expression and prognosis of HCC patients

**Fig. 3:**
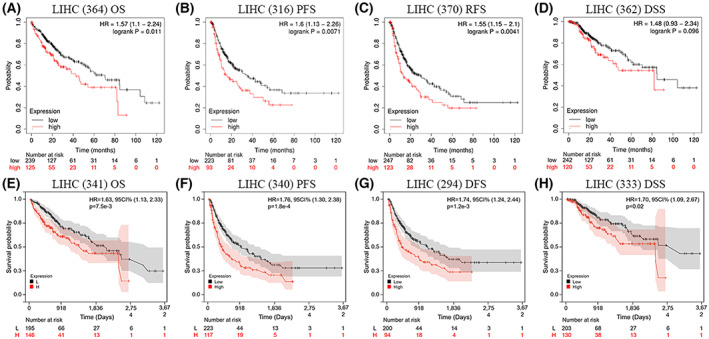
The correlation between high *RHBDF2* expression and prognostic value in HCC. (A–D) HCC patients with High *RHBDF2* expression had poor OS (*n* = 364), RFS (*n* = 316) and PFS (*n* = 370), but no DSS (*n* = 362) with the Kaplan–Meier plotter. (E–H) The correlation between *RHBDF2* expression and patient prognosis (OS, DFS, PFS and DSS) of HCC in The Cancer Genome Atlas database (http://cancergenome.nih.gov).

For an understanding of how RHBDF2 exerted influence on the prognosis of HCC patients, we first applied the Kaplan–Meier database for evaluating the link between RHBDF2 and prognosis in HCC patients. We observed that *RHBDF2* overexpression significantly correlated with poorer overall survival (OS) [hazard ratio (HR) = 1.57, *P* = 0.011], progression‐free survival PFS (HR = 1.6, *P* = 0.0071) and recurrence‐free survival (RFS) (HR = 1.55, *P* = 0.0041) in HCC patients, but not with disease‐specific survivial (DSS) (HR = 1.48, *P* = 0.096) (Fig. 3A–D). To evaluate the prognostic effect of RHBDF2, the clinical data of HCC were downloaded and a prognostic analysis of *RHBDF2* overexpression was performed. We found that the outcomes are consistent with the analysis of the Kaplan–Meier plotter (Fig. 3E–H). Therefore, these results implied that there is an obvious relationship between *RHBDF2* upregulation and worse prognosis in HCC.

**Fig. 4:**
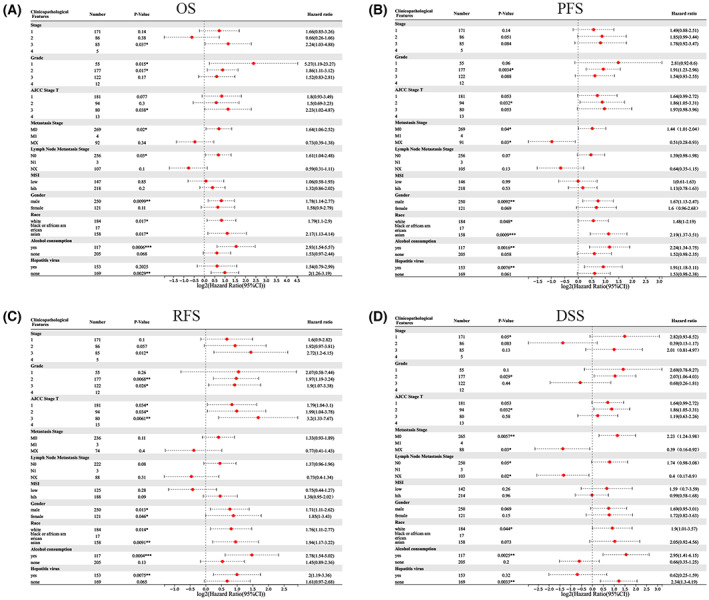
Forest plot of the prognostic values in various clinicopathological parameters of RHBDF2 in HCC. (A–D) Prognostic HR of RHBDF2 in different clinicopathological parameters in HCC for OS (A), RFS (B), PFS (C) and DSS (D). The significance was computed using the Cox–Mantel (log rank) test. *P < 0.05, **P < 0.01, ***P < 0.001.

The Kaplan–Meier plotter was employed for investigating the link between *RHBDF2* expression and different clinicopathological characteristics in HCC to further explore the prognostic function of RHBDF2 in HCC patients. For the tumor stage, *RHBDF2* overexpression was associated with unfavorable DSS in stage 1 HCC patients and related to bad OS and RFS in stage 3 HCC patients (Fig. 4A,C,D). In addition, *RHBDF2* upregulation was related to bad OS in American Joint Committee on Cancer (AJCC) stage T‐1 HCC patients, and related to unfavorable OS, bad RFS, poor PFS, poor DSS in AJCC stage T‐2 HCC patients, and associated with poor RFS in AJCC stage T‐3 HCC patients (Fig. 4A–D). For the vascular invasion, the results showed that *RHBDF2* upregulation was related to unfavorable RFS and PFS in HCC patients with no vascular invasion (Fig. 4B,C). For the tumor grade, *RHBDF2* overexpression was linked ed to unfavorable OS in stage 1 HCC patients and poor RFS in grade 3 HCC patients, and was related to worse OS, PFS, RFS and DSS in stage 2 HCC patients (Fig. 4A–D). For the metastasis stage, *RHBDF2* overexpression was related to unfavorable OS, PFS and DSS in HCC patients with M0, but related to good PFS and DSS in HCC patients with MX (Fig. 4A,C,D). For the lymph node metastasis stage, *RHBDF2* upregulation was linked to the bad OS and DSS in HCC patients with the N0 stage but related to good DSS in HCC patients with NX stage (Fig. 4A,C,D). Notably, the status of MSI does not influence the prognosis of HCC patients (Fig. 4). Moreover, a clear association between *RHBDF2* upregulation and worse OS and poor PFS was found in male HCC patients and poor RFS was observed in female HCC patients (Fig. 4A–C). Interestingly, a significant relationship between *RHBDF2* overexpression and worse OS, poor RFS, unfavorable PFS and worse DSS was found in white and Asian patients with HCC (Fig. 4A–D). Additionally, *RHBDF2* overexpression was clearly related to unfavorable OS, PFS, RFS and DSS in drinking HCC patients (Fig. 4A–D). Notably, we found that there is a noticeable link between RHBDF2 and worse RFS and PFS in HCC patients with a hepatitis history, and poor OS and DSS in HCC patients without a hepatitis history (Fig. 4A–D). Therefore, these results demonstrated that RHBDF2 has prognostic value in HCC patients.

### Mutation analysis of RHBDF2 in HCC

**Fig. 5:**
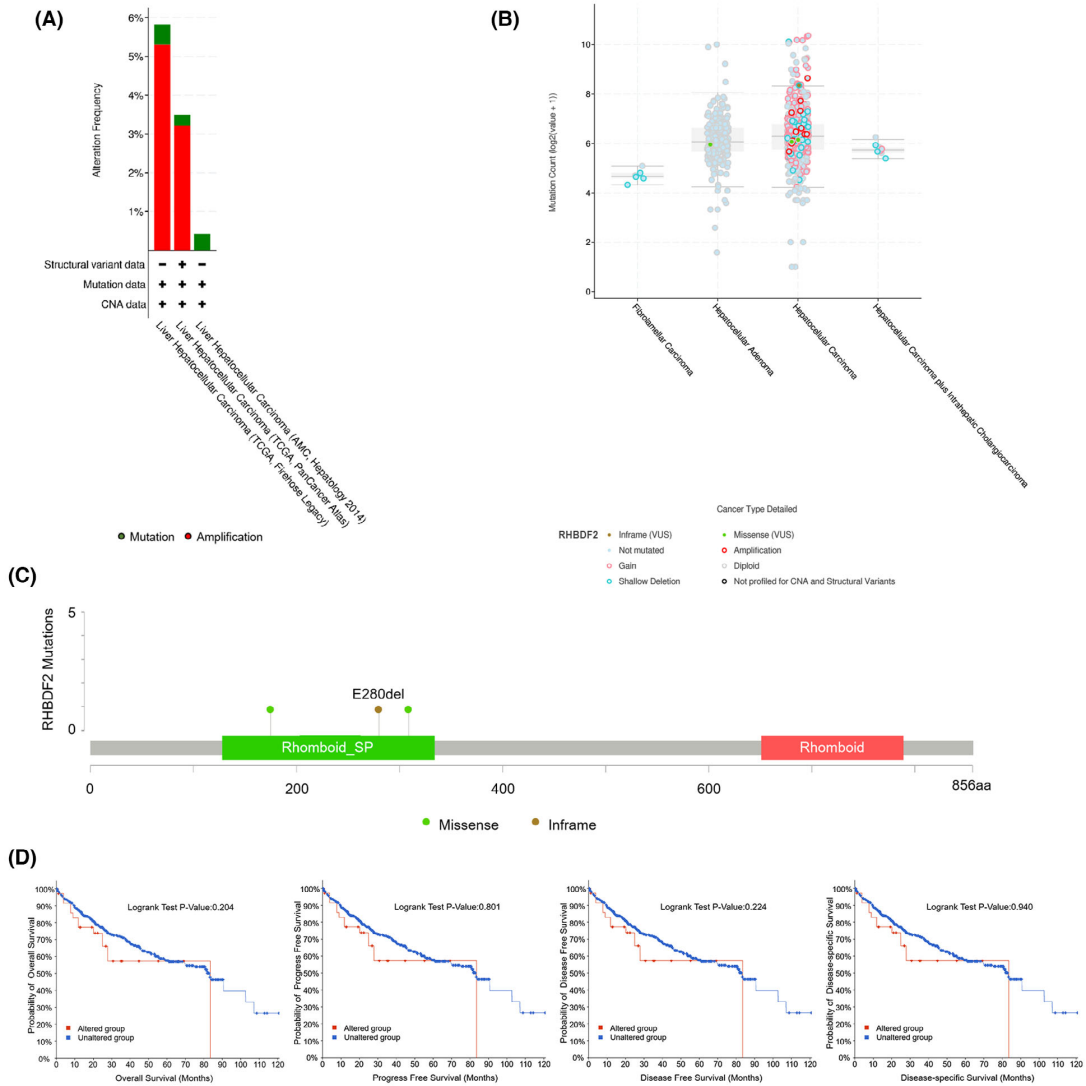
Alteration frequency of *RHBDF2* in HCC. (A) The alteration frequency of *RHBDF2* in HCC by the cBioPortal database. (B) The general mutation count of *RHBDF2* in HCC with the cBioPortal database. (C) Mutation diagram of *RHBDF2* in HCC across protein domains. (D) Kaplan–Meier plots were applied for predicting OS, PFS, DFS and DSS in cases with or without *RHBDF2* mutation.

To analyze the alteration frequency of *RHBDF2* in HCC, 1045 patients in total with HCC were investigated using the cBioPortal database (LIHC, AMC, Hepatology 2014; LIHC, TCGA, Firehose Legacy; LIHC, TCGA, PanCancer Atlas). The alteration frequency of *RHBDF2* was 5.84%, 3.49% and 0.43% in these three datasets, respectively (Fig. 5A,B). The most obvious type was amplification (Fig. 5A,B). We found four mutation sites (including three missense and one inframe mutation) between amino acids 128 and 334 (Fig. 5C). However, a statistical difference in OS, PFS, disease‐free survival (DFS) and DSS in HCC patients with or without RHBDF2 alterations was not observed with the Kaplan–Meier plotter (Fig. 5D).

### GO, KEGG and GSEA pathway analysis of RHBDF2 in HCC

**Fig. 6:**
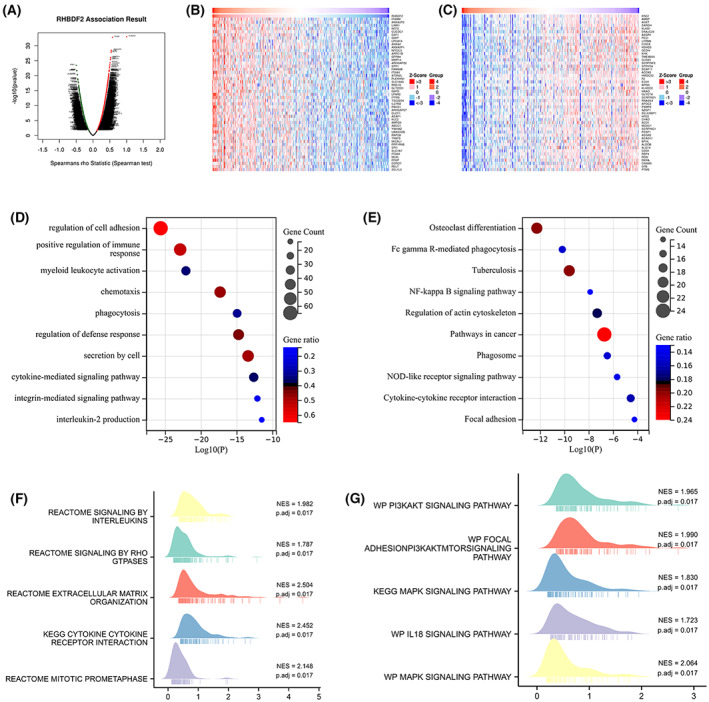
GO, KEGG and GSEA enrichment analysis of *RHBDF2* in HCC. (A) *RHBDF2* co‐expression genes identified by Pearson test in HCC. (B, C) The top 50 co‐expressed genes positively and negatively correlated with *RHBDF2* in HCC are shown. (D, E) Top 10 enrichment terms in GO categories (D) and KEGG pathways (E) in HCC. (F, G) GSEA enrichment terms of *RHBDF2* in HCC are shown.

Importantly, RHBDF2 co‐expressed genes were mined to evaluate the biological function of RHBDF2 in HCC using the LinkedOmics database (Fig. 6A). We showed the top 50 genes significantly correlated with *RHBDF2* (Fig. 6B,C). Then, the top 500 genes positively linked to *RHBDF2* were utilized for enrichment analysis of GO and KEGG to explore the biological value of RHBDF2. Subsequently, the top 10 enriched terms in GO and KEGG enrichment were obtained (Fig. 6D,E). Notably, GO biological process enrichment analysis indicated that RHBDF2 was related to some immune signaling pathways, including positive regulation of immune response, interleukin‐2 production and the cytokine‐mediated signaling pathway (Fig. 6D). Regarding KEGG pathway enrichment analysis, RHBDF2 was also associated with immune pathways, such as the TNF signaling pathway and cytokine–cytokine receptor interaction (Fig. 6E).

To obtain more insight into the molecular mechanism of RHBDF2 in HCC, GSEA analysis was carried out on the *RHBDF2* upregulation group and the *RHBDF2* downregulation group based on gene expression data sets. The enriched terms in KEGG (Fig. 6F,G) were also revealing. We also discovered that *RHBDF2* upregulation was related to multiple immune pathways in HCC according to KEGG pathway enrichment analysis. Therefore, the above results implied that RHBDF2 has important consequences in immune pathways in HCC.

### Association between RHBDF2 expression and immune infiltrates

**Fig. 7:**
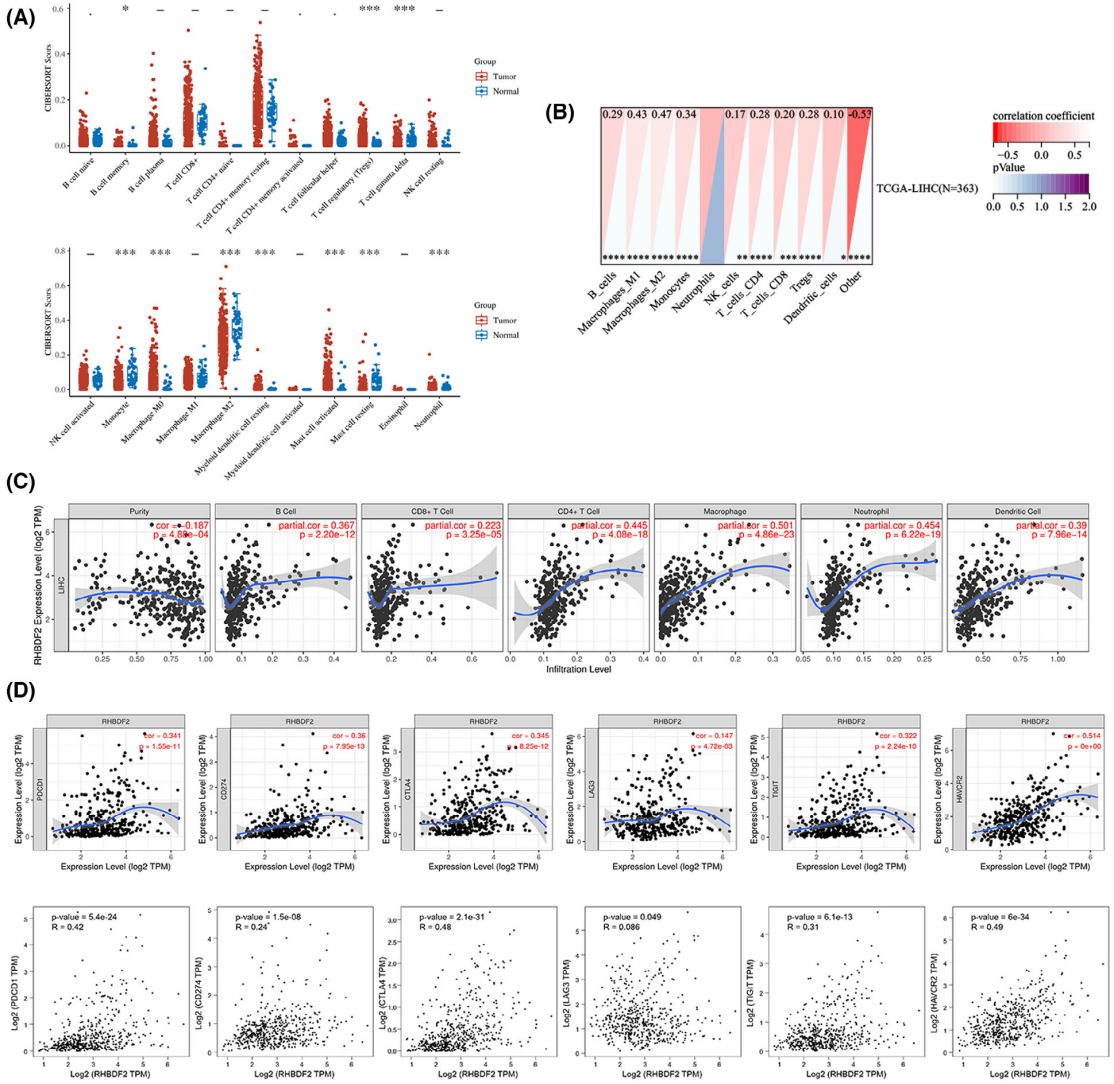
Correlation analysis of *RHBDF2* expression and immune infiltration in HCC. (A) *RHBDF2* expression has a significant association with the infiltration of immune cells in HCC using the CIBERSORT algorithm. (B, C) *RHBDF2* expression positively correlated with the infiltration of different immune cells using the quan TIseq tool and the TIMER database. (D) The positive link between *RHBDF2* expression and immune checkpoint expression in HCC using the TIMER database and GEPIA database.

Lymphocyte infiltration was an important independent predictor for relapse in HCC patients [28]. Therefore, 22 immune cell types in HCC and normal tissues were first analyzed with the CIBERSORT database. We discovered that immune cell infiltration was stronger in HCC compared to normal tissues (Fig. 7A). The Quan TIseq tool was utilized to investigate the association between *RHBDF2* upregulation and infiltration of varied immune cells in HCC. A significant relationship between *RHBDF2* upregulation and high immune cell infiltration was observed in HCC (Fig. 7B). Apart from this, the TIMER database was applied for evaluating the link between RHBDF2 upregulation and the infiltration of immune cells in HCC. The findings showed that the RHBDF2 upregulation was positively correlated with the infiltration of different immune cells in HCC. This was consistent with the analysis of the Quan TIseq tool (Fig. 7C). Cancer immune escape influences oncological outcomes and survival of cancer patients. Immune checkpoint molecules, such as PD‐1 and PD‐L1, can promote tumor cells to escape immune surveillance. To evaluate the association between RHBDF2 and the tumor microenvironment in HCC tissues, we investigated the link between *RHBDF2* and immune checkpoint genes. Notably, a strong correlation between *RHBDF2* overexpression and immune checkpoint gene upregulation was observed with the TIMER and GEPIA databases (Fig. 7D). These findings imply that RHBDF2 has important consequences for immune escape in the HCC microenvironment.

### Association between RHBDF2 expression and various immune markers

For a deeper understanding of the relationship between *RHBDF2* overexpression and immune infiltration in HCC patients, we evaluate the correlation between *RHBDF2* overexpression and gene markers of immune cells with the TIMER database. The outcomes showed that *RHBDF2* was clearly linked to most gene markers in HCC patients (Table 2). Additionally, the relationship between *RHBDF2* upregulation and gene markers of various functional T cells was assessed with the TIMER database. An obvious relationship between *RHBDF2* overexpression and gene markers of various functional T cells was shown with the TIMER database (Table 3). It is noteworthy that the association between *RHBDF2* expression and major histocompatibility complex (MHC) molecules was also investigated. We discovered that *RHBDF2* upregulation was clearly related to the MHC molecular markers, and this correlation had statistical significance (Table 4). These results implied that RHBDF2 has an essential impact upon immune cell infiltration of HCC.

**Table 2:** Association analysis between *RHBDF2* expression and gene markers of immune cells with the TIMER database.

Gene markers	None		Purity	
	Correlation	*P*	Correlation	*P*
B cell				
CD19	0.277	***	0.216	***
CD79A	0.239	***	0.175	**
CD8^+^ T cell				
CD8A	0.283	***	0.218	***
CD8B	0.178	***	0.108	*
Dendritic cell				
CD1C	0.231	***	0.159	**
HLA‐DPA1	0.433	***	0.387	***
HLA‐DPB1	0.405	***	0.349	***
HLA‐DQB1	0.281	***	0.212	***
HLA‐DRA	0.452	***	0.409	***
ITGAX	0.576	***	0.561	***
NRP1	0.482	***	0.46	***
M1 macrophage				
IRF5	0.538	***	0.548	***
NOS2	0.144	**	0.125	*
PTGS2	0.343	***	0.287	***
M2 macrophage				
CD163	0.385	***	0.332	***
MS4A4A	0.423	***	0.386	***
VSIG4	0.452	***	0.419	***
Natural killer cell				
KIR2DL1	0.042	4.17E‐01	0.013	8.15E‐01
KIR2DL3	0.205	***	0.212	***
KIR2DL4	0.249	***	0.218	***
KIR3DL1	0.113	*	0.099	6.66E‐02
KIR3DL2	0.123	*	0.084	1.21E‐01
KIR3DL3	0.071	1.75E‐01	0.053	3.30E‐01
KIR2DS4	0.103	*	0.097	7.27E‐02
Neutrophils				
CCR7	0.262	***	0.178	***
CEACAM8	−0.009	8.59E‐01	−0.036	5.03E‐01
ITGAM	0.641	***	0.624	***
Monocyte				
CD86	0.525	***	0.504	***
CSF1R	0.519	***	0.497	***
TAM				
CCL2	0.355	***	0.293	***
CD68	0.438	***	0.404	***
IL10	0.404	***	0.348	***
T cell (general)				
CD3D	0.28	***	0.224	***
CD3E	0.313	***	0.247	***
CD2	0.287	***	0.227	***

*P < 0.05, **P < 0.01, ***P < 0.001.

**Table 3:** Correlation analysis between *RHBDF2* expression and gene markers of different types of T cells with the TIMER database.

Description	Gene markers	None		Purity	
		Correlation	*P*	Correlation	*P*
Th1	IFNG	0.239	***	0.2	***
	STAT1	0.472	***	0.457	***
	STAT4	0.267	***	0.23	***
	TBX21	0.217	***	0.158	**
	TNF	0.387	***	0.358	***
Th1‐like	BHLHE40	0.31	***	0.302	***
	CD4	0.294	***	0.223	***
	CXCR3	0.32	***	0.259	***
	HAVCR2	0.514	***	0.491	***
	IFNG	0.239	***	0.2	***
Th2	STAT5A	0.479	***	0.438	***
	STAT6	0.401	***	0.413	***
Th17	STAT3	0.41	***	0.378	***
	IL17A	0.027	6.04E‐01	0.008	8.89E‐01
Treg	CCR8	0.478	***	0.457	***
	FOXP3	0.201	***	0.17	**
	TGFB1	0.478	***	0.452	***
Resting Treg	FOXP3	0.201	***	0.17	**
	IL2RA	0.442	***	0.402	***
Effector Treg T‐cell	CCR8	0.478	***	0.457	***
	FOXP3	0.201	***	0.17	**
	TNFRSF9	0.453	***	0.437	***
Effector T‐cell	CX3CR1	0.384	***	0.362	***
	FGFBP2	−0.075	1.47E‐01	−0.092	8.73E‐02
	FCGR3A	0.424	***	0.383	***
Naïve T‐cell	CCR7	0.262	***	0.178	***
	SELL	0.377	***	0.323	***
Effector memory T‐cell	DUSP4	0.404	***	0.361	***
	GZMA	0.179	***	0.112	*
	GZMK	0.193	***	0.109	*
Resting memory T‐cell	CD69	0.353	***	0.29	***
	CXCR6	0.268	***	0.198	***
	MYADM	0.463	***	0.45	***
General memory T‐cell	CCR7	0.262	***	0.178	***
	IL7R	0.349	***	0.294	***
	SELL	0.377	***	0.323	***
Exhausted T‐cell	CXCL13	0.134	**	0.106	*
	HAVCR2	0.514	***	0.491	***
	LAG3	0.147	**	0.096	7.39E‐02
	LAYN	0.382	***	0.338	***

*P < 0.05, **P < 0.01, ***P < 0.001.

**Table 4:** Correlation analysis between *RHBDF2* expression and gene markers of MHC molecules by with the TIMER database.

Description	Gene markers	None		Purity	
		Correlation	*P*	Correlation	*P*
MHC molecule	HLA‐A	0.408	***	0.378	***
	HLA‐B	0.304	***	0.278	***
	HLA‐C	0.316	***	0.316	***
	HLA‐DRA	0.452	***	0.409	***
	HLA‐DRB1	0.324	***	0.266	***
	HLA‐DQA1	0.378	***	0.334	***
	HLA‐DQA2	0.438	***	0.395	***
	HLA‐DQB1	0.281	***	0.212	***
	HLA‐DPA1	0.433	***	0.387	***
	HLA‐DPB1	0.405	***	0.349	***
	HLA‐DOA	0.445	***	0.406	***
	HLA‐DOB	0.317	***	0.263	***
	HLA‐DMA	0.404	***	0.362	***
	HLA‐DMB	0.441	***	0.405	***
	HLA‐E	0.405	***	0.379	***
	HLA‐F	0.247	***	0.211	***
	HLA‐G	0.264	***	0.217	***
	B2M	0.358	***	0.312	***
	TAP1	0.519	***	0.485	***
	TAP2	0.491	***	0.464	***
	TAPBP	0.494	***	0.476	***

*P < 0.05, **P < 0.01, ***P < 0.001.

### Prognostic analysis of RHBDF2 expression based on immune cells in HCC patients

The present study indicated that *RHBDF2* upregulation was surely related to immune cell infiltration in HCC. In addition, *RHBDF2* overexpression had an unfavorable prognosis for HCC patients. Thence, we conjecture that RHBDF2 can impact the prognosis in HCC patients partly because of immune cell infiltration.

**Fig. 8:**
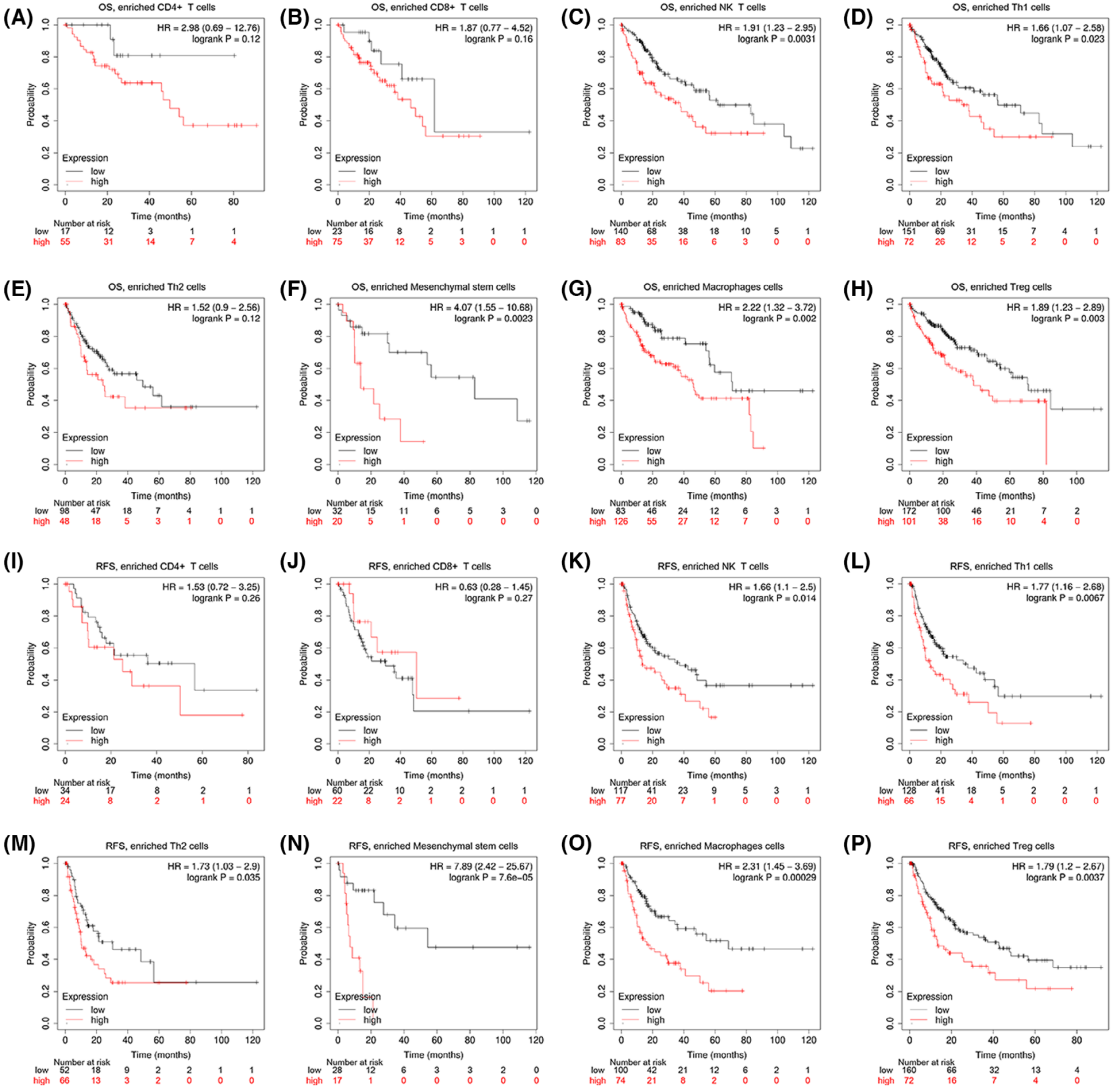
Kaplan–Meier survival curves of high and low expression of *RHBDF2* based on immune cells subgroups in HCC. (A–H) HCC patients with high *RHBDF2* expression and enriched natural killer T cells, Th1 cells, mesenchymal stem cells, macrophages and regulatory T cells had a poor OS. (I–P) Correlations between *RHBDF2* expression and RFS in different immune cell subgroups in HCC patients were analyzed by Kaplan–Meier plotter.

The Kaplan–Meier plotter was applied for performing the prognosis evaluation of RHBDF2 based on immune cell infiltration subgroups in HCC. The results showed that patients with high *RHBDF2* expression and enriched natural killer T cells (*P* = 0.0031), Th1 cells (*P* = 0.023), mesenchymal stem cells (*P* = 0.0023), macrophages (*P* = 0.002) and regulatory T cells (*P* = 0.003) had a poor OS (Fig. 8C,D,F–H). However, there is no link between *RHBDF2* overexpression and OS in HCC patients with enriched CD4^+^ memory T cell, CD8^+^ T cell and Th2 cell infiltration (Fig. 8A,B,E). Similarly, high *RHBDF2* expression in HCC patients was associated with a worse RFS in enriched natural killer T cells (*P* = 0.014), Th1 cells (*P* = 0.0067), Th2 cells (*P* = 0.035), mesenchymal stem cells (*P* = 7.6E‐05), macrophages (*P* = 0.00029) and regulatory T cells (*P* = 0.0037) (Fig. 8K–P). However, we found no relationship between *RHBDF2* expression and RFS in HCC patients with enriched CD4^+^ memory T cells and CD8^+^ T cells (Fig. 8I,J). The above results implied that RHBDF2 may have important consequences in the prognosis of HCC patients partly because of immune infiltration.

## Discussion

HCC is a primary malignancy and its mortality and morbidity is increasing year‐to‐year [4, 29, 30, 31]. Surgical treatment is the first choice for early‐stage HCC patients, and chemotherapeutic drugs, radiotherapies, targeted therapies and immunotherapy are suitable for advanced patients. However, effective treatments for advanced HCC are lacking [4, 7, 8, 32, 33]. Thus, it is important to explore a new prognostic biomarker in HCC urgently. *RHBDF2* is a protein‐coding gene that participates in important biological processes, consisting of regulation of ADAM17, TNF signaling, and EGFR activation [34, 35]. For a detailed understanding, the biological roles and potential mechanism of RHBDF2 in HCC, as well as the expression, prognosis and mutation of *RHBDF2*, and its link with immune infiltrates in HCC, were first analyzed.

In the present study, significant *RHBDF2* overexpression in HCC tissues was discovered using the TIMER, GEPIA, and UALCAN databases compared to normal tissues. These results indicated *RHBDF2* was correlated with progression and had a pro‐cancer influence in HCC. Subsequently, we further investigated *RHBDF2* expression in HCC cell lines and HCC tissues. Immunohistochemical staining was utilized to investigate *RHBDF2* expression in HCC tissues. Additionally, RT‐qPCR and western blotting were employed for the evaluation of *RHBDF2* expression in HCC cell lines. These outcomes indicated that *RHBDF2* was remarkably upregulated in HCC cell lines and HCC tissues.

Additionally, we also assessed the link between RHBDF2 and clinicopathological parameters and found that *RHBDF2* overexpression was significantly linked to tumor histology, stage, lymph node metastasis and TP53 mutation. These results are consistent with previous research. High *RHBDF2* expression was positively associated with tumor stage, tumor size and extent of tumor invasion [23]. In addition, *RHBDF2* alteration frequency in HCC was investigated with the cBioPortal database. The results suggested that HCC patients had alterations of *RHBDF2*, and the most obvious type was amplification. From the results of survival analysis, *RHBDF2* upregulation was clearly related to worse OS, RFS and PFS, which is consistent with previous findings suggesting that RHBDF2 has a crucial influence on poor prognosis [23]. Our results implied that *RHBDF2* was linked to the prognosis in HCC patients. Furthermore, we further researched the link between *RHBDF2* and prognostic value in different clinicopathological parameters of HCC patients. Multivariate COX regression analysis suggested that *RHBDF2* is a risk factor, which was consistent with survival analysis. Thus, these results implied that *RHBDF2* can be a novel potential biomarker for the prediction of prognosis in HCC patients.

Immune escape and immune tolerance have important influences on tumor progression and immune infiltration is linked to tumor occurrence, development and metastasis as a result of HCC being a typical inflammation‐related tumor [36, 37, 38]. Therefore, a comprehensive analysis of immune infiltrates in HCC patients was first carried out to reflect the changes in immune status in HCC patients. For CIBERSOT analysis, the infiltration of 22 immune cells was clearly increased compared to normal tissues. Previous studies have reported that RHBDF2 was involved in the immune process of disease. For example, the upregulation of *RHBDF2* expression can promote the progression of cervical cancer by inducing TGF‐β transcription [23], RHBDF2 promotes the transport and activation of ADAM17 is the key to activating tumor necrosis factor signaling [35], RHBDF2 can mediate inflammation by releasing TNF‐α [22, 39, 40, 41, 42] and downregulation of *RHBDF2* expression can lead to changes of cytokine production in T helper cells [43]. Therefore, to evaluate the potential biological values of RHBDF2 in HCC, *RHBDF2* co‐expressed genes were applied for enrichment analysis of GO and KEGG. We found that *RHBDF2* co‐expressed genes were related to multiple pathways of the immune process with GO and KEGG enrichment analysis, which was consistent with the GSEA analysis. In many cancer patients, the lymphocyte infiltration rate can independently predict survival rate and lymphatic metastasis [44]. Lymphocyte infiltration rate predicts the recurrence in HCC patients [28]. Therefore, the link between RHBDF2 and immune infiltration was evaluated through correlation analysis, and *RHBDF2* upregulation was likely related to multiple immune cell infiltration in HCC. Additionally, a strong correlation between *RHBDF2* upregulation and immune checkpoint genes (PDCD1, CD274, LAG3, CTLA4, TIGIT, HAVCR2) was observed in HCC, and these immune checkpoint genes can contribute to tumor immune escape and promote tumor progression [45, 46, 47]. It is noteworthy that *RHBDF2* upregulation was clearly associated with various immune cell markers, lymphocyte subset markers and MHC molecular markers in HCC. Therefore, the present study showed that *RHBDF2* overexpression was indeed linked to the immune infiltrates in HCC patients, which indicated that RHBDF2 might have a crucial influence on the immune infiltration of HCC.

More importantly, we observed that *RHBDF2* overexpression in HCC with enriched immune cells had a worse prognosis through the analysis of the Kaplan–Meier plotter. Thus, RHBDF2 can impact the prognosis of HCC patients partly because of immune infiltration. The high density of Tregs is positively associated with vascular invasion and negatively related to tumor packaging [48] and the high density of Tregs is linked to the worse prognosis of HCC patients [49]. Macrophage promoted tumor growth and invasion and predicted poor prognosis in HCC [50, 51, 52, 53, 54, 55, 56]. These outcomes indicated that *RHBDF2* can act as a new prognostic biomarker related to immune infiltration of HCC.

The present study can strengthen our comprehension of the function of RHBDF2 in HCC; that is, up‐regulated *RHBDF2* is clearly linked to clinicopathological features, an unfavorable prognosis and immune infiltrate in HCC, although there are still some limitations. First, most of the investigation is based on the mRNA level of *RHBDF2*. An in‐depth analysis based on the protein level is more convincing. Second, RHBDF2 may have an essential impact upon the immune regulation process of HCC, but its molecular mechanism needs more research. Overall, our findings implied that *RHBDF2* overexpression can predict the worse prognosis of HCC patients. Furthermore, our research implied that *RHBDF2* upregulation was significantly linked to immune cell infiltration and revealed a potential molecular mechanism by which that RHBDF2 can impact the prognosis of HCC patients partly because of tumor immune infiltration.

## Conclusions

The present study systematically analyzed the expression, prognosis and immune infiltrates of RHBDF2 in HCC. It was implied that *RHBDF2* overexpression was clearly linked to the unfavorable prognosis of HCC patients partly because of immune cell infiltration. Therefore, we concluded that *RHBDF2* could be a new biomarker for the prediction of the prognosis of HCC patients.

## Conflicts of interest

The authors declare that they have no conflicts of interest.

## Author contributions

HG conceived and designed the study. HG, ZH, SZ and HX carried out the acquisition of data. HG, HX and ZH took part in cell culture and RNA extraction. HG, YT, MX and ML analysed the data. HG wrote the manuscript. YW revised the manuscript and supervised the project. All authors contributed to the article and approved the final version submitted for publication.

## Data Availability

The datasets reported in the present study can be found in online repositories. The names of the repository/repositories and accession number(s) are provided within the article itself.
